# What Do Clinicians Who Deliver Maternity Services Think Patient-Centered Care Is and How Is That Different for Vulnerable Women? A Qualitative Study

**DOI:** 10.1155/2018/5853235

**Published:** 2018-12-02

**Authors:** Ted Adams, Dana Sarnak, Joy Lewis, Jeff Convissar, Scott S. Young

**Affiliations:** ^1^MBChB MRCOG MSc, Consultant in Obstetrics and Gynaecology, Southport and Ormskirk NHS Trust, Wigan Road, Lancashire L39 2AZ, UK; ^2^MPH, Research Associate, Commonwealth Fund of New York, 1 East 75th Street, New York, NY 10021, USA; ^3^MSW MPH, Senior Health Policy Consultant, Institute for Health Policy, Kaiser Permanente, Washington, DC, USA; ^4^MD, Medical Director Care Management Institute, Kaiser Permanente, Oakland, CA 94612, USA; ^5^MD, Executive Director & Senior Medical Director, Care Management Institute, Kaiser Permanente, Oakland, CA 94612, USA

## Abstract

**Background:**

Patient-centered care is said to have a myriad of benefits; however, there is a lack of agreement on what exactly it consists of and how clinicians should deliver it for the benefit of their patients. In the context of maternity services and in particular for vulnerable women, we explored how clinicians describe patient-centered care and how the concept is understood in their practice.

**Methods:**

We undertook a qualitative study using interviews and a focus group, based on an interview guide developed from various patient surveys focused around the following questions: (i) How do clinicians describe patient-centered care? (ii) How does being patient-centered affect how care is delivered? (iii) Is this different for vulnerable populations? And if so, how? We sampled obstetricians and gynecologists, midwives, primary care physicians, and physician assistants from a health management organization and fee for service clinician providers from two states in the US covering insured and Medicaid populations.

**Results:**

Building a relationship between clinician and patient is central to what clinicians believe patient-centered care is. Providing individually appropriate care, engaging family members, transferring information from clinician to patient and from patient to clinician, and actively engaging with patients are also key concepts. However, vulnerable women did not benefit from patient-centered care without first having some of their nonmedical needs met by their clinician.

**Discussion:**

Most providers did not cite the core concepts of patient-centered care as defined by the Institute of Medicine and others.

## 1. Introduction

There is potential for a misunderstanding between patients, providers, and policy makers of what patient-centered care is. This study sets out to understand what health care professionals delivering maternity services understand by the term patient-centered care. Our focus is on how providers delivering patient-centered care believe they enhance care for all women, and in particular, vulnerable women. We used* The Commonwealth Fund's *[1] definition of vulnerability. It includes people without health insurance, low-income families, and racial and ethnic minorities.

The concept of patient-centered care has gained traction in health care organizations over the last few years. Evidence shows that practicing elements of patient-centered care can produce favourable outcomes such as reduction in the utilization of health care services and related costs [2], improvement in patient and family satisfaction with care [3], increased patient adherence to medication regimen, and a reduction in decisional conflict between patients and clinicians [4]. Authors who describe patient-centered care include the provision of health promotion and self-care as part of the process of delivering patient-centered care [5–8].

The heterogenous effects of patient-centered care may be related to the lack of a generalized definition of patient-centered care. The Institute of Medicine's frequently cited definition says it “entails medical care processes that ensure decisions regarding the care received respects each patient's wants, needs and preferences, and for which the patient has the education and support he or she needs to make decisions and participate in his or her own care” [9]. The Institute of Patient and Family-Centered Care, which is a US not for profit organization, sets out four core constructs: respect and dignity, information sharing, participation, and collaboration [10].

Understanding how professionals approach the concept of patient-centered care in maternity services may help us to ensure that clinicians and policy makers, who cite the benefits of patient-centered care, understand each other's perspectives. It may also help organizations to commission or deliver maternity care to vulnerable women more effectively and in doing so improve the quality of care they offer to all pregnant women. Our research questions were

- How do clinicians describe patient-centered care?
- How does being patient-centered affect how care is delivered?
- Are the answers different for vulnerable populations? And if so, how?

## 2. Methods

In 2013 Childbirth Connection (now part of the National Partnership for Women and Families) released its* Listening to Mothers 3* [11] report. This report investigated women's views on how maternity care was delivered across the geographical United States. The authors were careful to represent all demographics in the survey. We developed a qualitative interview guide using

- Listening to Mothers 3,
- NHS choices [12], a freely available patient comment, rating and information website run by the UK's National Health Service,
- Kaiser Permanente's HCAHPS [13] survey responses from 2008 to 2014.

We used a qualitative approach to explore what clinicians think patient-centered care is and whether it is different for vulnerable patients. We wanted to draw out whether there were differences in what patient-centered care meant when treating different patients, in terms of health status, health care coverage, income, area of domicile, socioeconomic status, and racial profile. We chose qualitative investigation in preference to quantitative methods as qualitative study is more suited to understanding the beliefs, qualities, and motivations of professionals [14].

We wanted to capture the potentially diverse views of different maternity service clinicians across various types of organizations. We used a purposive sampling approach. This also increased transferability of our results. We spoke to 16 individuals, undertaking 13 interviews and one focus group, lasting between 40 and 90 mins. The interviews were semistructured and followed a written interview guide. The interview guide was piloted and received specific IRB approval. The guide was divided into three main sections. We asked clinicians to explain

1. What gave them joy at work.
2. What they thought patient-centered care was and their opinion of the construct.
3. We then asked them to expand their thoughts on patient-centered care in the light of a clinical scenario whereby a woman requested a Caesarean section.

At the end of each section, we prompted the clinicians to say whether and how their answers would be different for a vulnerable woman. This paper reports on the latter two sections.

We planned to speak to obstetricians, midwives, family physicians, and physician's assistants across 9 sites in California and Virginia that were chosen to represent the diversity of women treated by providers. We originally approached 15 sites for interviewees. The basic demographic data of our participants is available in Table 1. Nine sites provided at least one person for interview; six sites did not. After agreeing to be interviewed, no participants refused to participate; however one site withdrew prior to interview because of illness. No participants withdrew after interview. All the interactions were face to face and undertaken by the same person in the interviewee's office or home. No other person was present during the interviews. The interviewer had undergone training with The Commonwealth Fund prior to undertaking the first interview. The interviews were carried out between January and May 2015. During the interview, the interviewer's position as a visiting obstetrician from outside the country was stressed with the aim of enhancing the interviewer's credibility and encouraging candid response. Field notes were taken during the interview.

The organization types involved were a large managed care organization and two smaller fee-for-service physician groups. In California, the population covered by the clinicians we interviewed was mixed, ranging from the affluent areas of the Napa Valley to the less affluent areas of the California Central Valley. In Virginia, the populations were mainly a mixture of women covered by private insurers and Medicaid (the U.S. government program, financed by federal, state, and local funds, of hospitalization and medical insurance for persons of all ages within certain income limits).

The interviews were recorded and professionally transcribed verbatim. The transcripts were read before being coded using an inductive approach by two separate coders, using Atlas.ti® v7 (Berlin, Germany). One coder (TA) was the interviewer; the other coder was a nonclinician with experience of the social sciences and qualitative research (DS) to improve triangulation [15]. A preliminary code book was developed independently by each coder after the first two interviews. The transcripts were then reviewed by both coders and each code was systematically discussed with the other coder to ensure congruence in the development of the code book. These steps were performed each time after two interviews had been transcribed. After transcript six, the code book was sense-checked by JC. The systematic discussion after each transcription continued to ensure that saturation was achieved. The codes were thematically analyzed and then relationships between the codes were defined using linkages and co-occurrences between codes. The study was approved by the Northern California Kaiser Permanente IRB.

## 3. Results

In discussing patient-centered care, clinicians described three key themes. The three themes are presented below:

1. Building a trusting relationship between the clinician and the woman is fundamental to patient-centered care.
2. Patient-centered care involved individualizing care for women and their families.
3. Patient-centered care meant that clinicians had to engage with and involve women and their families.

### 3.1. Building a Trusting Relationship

Building a relationship between clinicians and women is central to what clinicians believe patient-centered care is.

“Investing in the beginning and really creating [a] relationship with my patients is extremely valuable” Obstetrician

The familiar parts to any interaction were considered part of building a trusting relationship, for example, clinicians being committed to their patients in terms of time, active listening, and giving undivided attention.

“it really is about that one-on-one and that focusing and, you know, sitting down right in front of them and being there.” Obstetrician

Trust from the perspective of the clinician was very much related to the information that flowed from clinicians to women.

“it always feels good to me when I know that a patient understands … the information that I'm trying to give her” Obstetrician

There was also an understanding of the complexity of developing a trusting relationship and that whilst the main issue for clinicians was information transfer, how women received and processed that information would affect the development of the relationship. If a trusting relationship was to be established, some clinicians acknowledged that this requires trust on the part of women too.

“So, it's a balance between having an idea and vision for what they want and giving me a little bit of trust in knowing that I've done this many times and that I'm not out to foil their plan” Midwife

### 3.2. Individually Appropriate Care

Giving individually appropriate care is very important to providing care that is patient-centered.

Individual care is described by our participants as giving medically and socially appropriate care to women. That is not synonymous with care that the woman requests directly. If a woman were to ask for care that the clinician did not believe was in her best interests then all of the clinicians we spoke to would have reservations about providing the requested care. All the clinicians would discuss the options with the woman, with some refusing to provide the requested care. Some clinicians would acquiesce to the request but it would depend on what the request was. For example, some clinicians would provide a Caesarean section on request; others would not. None of the clinicians believed that patient-centered care meant that a patient could demand care that was not suited to that particular woman.

“on an individual basis, I think patient-centered care involves … humanizing the patient, and then getting … what she needs out of the situation.” Obstetrician

“care that suits their needs, that is appropriate for them, medically. But also, I guess, socially or socioeconomically” Obstetrician

“I think elective Caesarean.. by maternal request for no indication,..I think that's bad medicine…So, on that one,.I don't think patient-centered care is about providing poor care.” Midwife

Individualizing care in labour was frequently cited as an example of where patient-centered care could be put into practice. There was an understanding that women's perceptions of their individual intrapartum experience were central to being patient-centered.

“a good birth is a birth where the woman feels like she was listened to and that she thought it was a good birth – not that I necessarily thought it was a good birth; that she thinks it's a good birth…that's patient-centered care” Midwife

Clinicians were aware of the difficulty that intrapartum uncertainty created for both women and professionals. This uncertainty could create tension between the best care that clinicians wanted to deliver and what women believed was for the best, clinicians being cognisant that sometimes the evidence for any approach was grey with the result that being patient-centered can leave women and clinicians unfulfilled.

“You can have patient-centered care, but still have everyone sort of unhappy about it. So you know, someone that comes in and refuses, like, refuses monitoring at all, makes everybody nervous.” Midwife

### 3.3. Engaging Women and Their Families

Engaging with family and women's support networks was thought to be very relevant in delivering patient-centered care. Clinicians saw that they had to interact with families at many different levels. If clinicians were to be patient-centered they had to get an idea from women how family members should be involved in their care. Family members were part of a much more complex group that could support and influence women positively and negatively.

“a way of being patient-centered and involving the family members is identifying from the patient what role she wants her family members to play, and then facilitating that role.” Family Physician

Clinicians said that if they were to deliver the best care to women they also needed to have women's families positively involved in the therapeutic relationship. At the other end of the spectrum there was also the danger of families negatively affecting the care that women might receive.

“Sometimes we have people's partner [who] doesn't want them to get pain relief… and I'll just say, “You know, it really needs to be her answer,” you know?” Midwife

It should be noted that these three themes were not mutually exclusive. For example, understanding that women needed individualized information was part of relationship building and providing individualized care. Individualizing care also involved building relationships and understanding the effect of an individual's support network. There was surprising congruity between clinician types in terms of most responses, with the main difference being that those clinicians in fee for service found it harder to refer to services which they did not directly provide.

“I mean, we – I had no help on that end [psychiatric care]. And I didn't know who to contact…they [insurance companies] don't tell us, “If you have a patient that has this, this is where you go.” We don't have that.” Physician assistant

### 3.4. How Is Patient-Centered Care Different for Vulnerable Populations?

Most clinicians said categorically that they would not treat a vulnerable woman differently from someone whom we did not define as vulnerable. However as each interview progressed, all clinicians qualified their initial response, most finding a reason why vulnerable women would in fact be treated differently;

“my assessment isn't different, the answers are often different” Family Physician.

Our respondents agreed that delivering patient-centered care to this group was more challenging because of their increased nonmedical needs. This often had the effect of lessening the ability of the clinician to fully engage with vulnerable groups in shared-decision making as nonmedical needs were more likely to be of a higher priority.

“Some of the issues that I want in their care might be a little different, because my focus might be more on…`So how is it going not smoking marijuana'?" Midwife

Our results showed that clinicians dealt with this concept by developing the relationship between them and women.

“I think their patient-centered care is a little bit different. I think it's a little more intense. I think they need more one-on-one. They need more hand-holding. They need more navigating through our system… So it's about educating them. It's not educating them because they're dumb; it's educating them because they don't have – they don't have the background. They don't have the information. They don't have the tools.” Obstetrician

Women's families were important in helping vulnerable women with their interaction and understanding of maternity care.

“I have a patient who's a teenager right now. She lives with her grandmother… I'm trying to encourage her to attend classes,.. And her grandmother accompanies her to these visits. So, the two of us will tag-team in helping her understand that there are certain things that you have to be ready for.” Obstetrician

It was also clear that meeting basic nonmedical needs had to be achieved or at least addressed before clinicians could move on to shared decision-making. These needs were often simple yet were acknowledged by clinicians as real barriers in delivering patient-centered care. Clinicians felt that they were in a position to overcome nonmedical needs, for example, by persuading their organization to provide free transport to facilities or changing appointment times to fit local large employers shift patterns.

“..not everybody in the family has a car. They're taking two buses to come visit me.” Obstetrician.

Meeting nonmedical needs allowed the relationship to further develop and in doing so, there is improved understanding, on the clinician's side, of the difficulties individual women have and concurrently a better understanding of how to improve the personal health of the individual woman:

“[improving the health of vulnerable patients] I think, takes more conversations, because part of it is their understanding or their literacy of their trust of doctors and hospitals, etc. So I think you've got to build that trust first.” Obstetrician

## 4. Discussion

### 4.1. Main Findings

Our study shows that clinicians believed developing a relationship with their patients is central to their understanding of patient centred care. The relationship was developed through information transfer and trust and understanding and involving women and their family in their medical care. For vulnerable women, clinicians showed that they have to develop an understanding of the nonmedical issues faced by their patients and the effect of this on their medical care in order to deliver patient-centered care.

### 4.2. Strengths and Limitations

This study is qualitative in its design, which encourages a breadth of information collection. The authors ensured that the different types of clinicians, from differing types of care provider (e.g., fee for service, managed care) who provide maternity care, were represented in the study. The study was designed from the outset using COREQ guidelines [16]. The clinicians we spoke to were reassured around their anonymity and appeared to speak freely; indeed most spoke passionately about their commitment to delivering how they described patient-centered care.

The study was completed in the United States only. Patient-centered care as a concept has travelled across the world, with a recent BMJ spotlight, citing evidence about patient-centered care from Australia, the UK, India, and the US [17]. The study's applicability across different health care systems may be questioned; however, the results we obtained seem applicable to all professionals, mainly because of the coherence that we achieved from the different types of clinician. Coherence is a better test for qualitative studies than whether a study is representative [18]. The study's initial timescales and sampling sites were constrained by the length of the fellowship and the funding available for travel across the US; however, data saturation was achieved within this funding and time envelope.

### 4.3. Interpretation

When one compares the importance of the clinical relationship to clinicians with the core concepts of patient-centered care as defined by the Institute of Medicine or The Institute of Patient and Family Centered Care, there is a difference in shared understanding. Kiston et al. [19] discuss how groups of health care professionals understand patient-centered care differently. Clinicians' responses tended to be focused on the woman whom they imagined in the consultation in front of them and were focused mainly in terms of the relationship they develop with the women they serve. Stewart et al.'s [20] method of patient-centered care appears closest to the description we heard. Most clinicians did not cite any of the core concepts of patient-centered care as defined by the Institute of Medicine or The Institute of Patient and Family Centered Care directly, although there was congruity and some common areas, for example, the theme of respect for patients, were clearly in evidence. Clinicians always prioritized good relationships between them and patients, and whilst some of the core concepts of patient-centered care would be impossible without a good relationship, the primacy of the relationship and the fact that developing a good relationship is not called out as core to the concepts may have the effect of alienating some clinicians from engaging with the described concept of patient-centered care.

Pregnant women are generally not unwell and require different forms of support during pregnancy compared to other health care populations. De Labrusse et al.'s [21] systematic review sets out how patient-centered care in maternity services is different from other populations. They describe how the two most important differences are because care is delivered under normal physiologic conditions as well as pathology and that caregivers are multidisciplinary.

Our results suggest that vulnerable pregnant women create a further difference that may be generalizable to vulnerable men and vulnerable nonpregnant women. Clinicians in our study understood that “people are more than their diseases and that their social relationships, housing conditions, and economic status can affect their disease trajectories and care choices” [22]. These potential vulnerabilities affect the way in which patient-centered care is delivered and need acknowledging and preferably ameliorating before clinicians are able to address a patient's medical needs. A clinician must try to engage with an individual's nonmedical needs in order to enhance the relationship.

**Figure 1:**
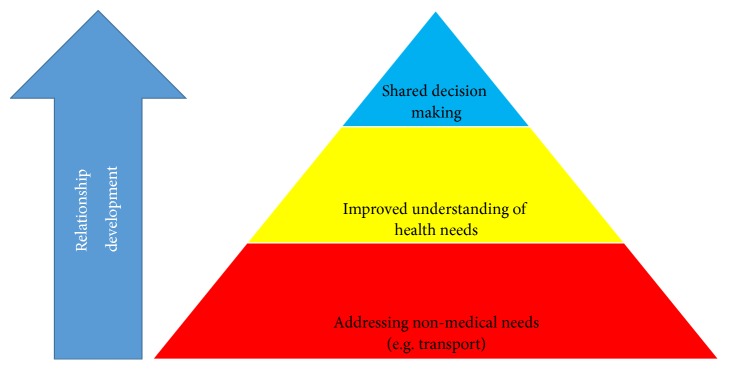
If nonmedical needs are met or at least acknowledged there can be better understanding of health needs, which in turn leads to easier shared decision-making. Relationship development will facilitate each transition.

We have therefore proposed a triangle of patient-centered care (Figure 1), based on an interpretation of Maslow's hierarchy of needs [23]. In our model, vulnerable women's nonmedical needs are met prior to their medical needs, for example, by improving accessibility to medical services. Each tier within our model blends into one another, but there is a hierarchy. At the top of the triangle is more of a willingness from clinicians to engage in shared-decision making. As nonmedical needs are at the very least acknowledged and trust builds between clinician and woman, shared decision-making becomes easier for both woman and clinician.

In our study, clinicians state that the relationship between clinicians and women is the foundation of patient-centered care. Promotion of patient-centered care may mean moving away from thinking in terms of numbers of interactions between women and clinicians and instead looking at the quality of interactions. Measuring the quality of the clinician–patient relationship is acknowledged as difficult [24]. This ties in with the acknowledged difficulties in measuring how elements of patient-centered care are provided [25]. To some extent the medical interaction rating websites, for example, NHS Choices [12], have begun to measure the quality of relationships; however, the comments on ratings websites might not be reflective of all clinician–patient exchanges that are undertaken by a clinician or facility. Some domains of existing patient questionnaires already touch on elements of the clinician–patient relationship, for example, the communication composite scores in HCAHPs [13]. Patient reported outcome measures (PROMs) have been used in health care for some time; however, there is evidence that the output from some PROMs does not always influence patient care [26]. Chow et al. [27] examined the concept of PROMs and discussed the often missed importance of measuring patient satisfaction when thinking about which outcome measures are most suited to particular areas. They go on to say that “measurement of patient satisfaction itself has taken a back seat to quality of life and assessment of current health state.” Our research supports this and adds weight to the argument that careful interrogation and understanding of the relationship could mirror patient satisfaction and that clinicians would value patients' views on the relationship they have with their health care professional.

## 5. Conclusion

It would be difficult for anyone in health care, be they patients, professionals, or policy makers, not to accept prima facie the idea of patient-centered care. However, a mixed understanding between professionals and policy makers of what patient-centered care is creates diverging beliefs on what its benefits are. We maintain that continuing efforts to develop a way of measuring clinician–patient relationships, trusted by both clinician and patient that did not interfere with the relationship, would be a way of promoting patient-centered care. This measurement should be based around patient and clinician satisfaction with the relationship. Policy makers do not always look towards building trust and connections between clinicians and patients despite the obvious benefits of doing so. We maintain that the centrality of the clinician-patient relationship to the concept of patient-centered care must be made more explicit to clinicians and policy makers in order for the benefits of patient-centered care to be fully realized.

## Figures and Tables

**Table 1:** Participants' demographics and other relevant information.

Sex	Female 14; Male 2
Type of care provided	Fee for service practitioners 5 Managed care practitioners 11

Professional group	Obstetricians 10; Family practitioners 1; Midwives 4; Physician's assistants 1

Location	West coast 11; East coast 5

Incentive to participate	none

Recruitment	By email invitation. Email approved by IRB

Eligibility	Fully qualified, not in training, in current clinical practice, responsible for the delivery of a baby within the last 12 months or contributed significantly to the antenatal or postnatal care of a pregnant woman within the last 12 months.
